# Adherence to the 2015 Dutch dietary guidelines and risk of non-communicable diseases and mortality in the Rotterdam Study

**DOI:** 10.1007/s10654-017-0295-2

**Published:** 2017-08-19

**Authors:** Trudy Voortman, Jessica C. Kiefte-de Jong, M. Arfan Ikram, Bruno H. Stricker, Frank J. A. van Rooij, Lies Lahousse, Henning Tiemeier, Guy G. Brusselle, Oscar H. Franco, Josje D. Schoufour

**Affiliations:** 1 Department of Epidemiology, Erasmus MC, University Medical Center, Rotterdam, The Netherlands; 2 Leiden University College, The Hague, The Netherlands; 3 Department of Internal Medicine, Erasmus MC, University Medical Center, Rotterdam, The Netherlands; 4 Department of Respiratory Medicine, Erasmus MC, University Medical Center, Rotterdam, The Netherlands; 5 Department of Respiratory Medicine, Ghent University Hospital, De Pintelaan 185, 9000 Ghent, Belgium; 6 Department of Psychiatry, Erasmus MC, University Medical Center, Rotterdam, The Netherlands

**Keywords:** Diet quality, Validation, Cardiovascular disease, Cancer, Neurological diseases, Cohort study

## Abstract

We aimed to evaluate the criterion validity of the 2015 food-based Dutch dietary guidelines, which were formulated based on evidence on the relation between diet and major chronic diseases. We studied 9701 participants of the Rotterdam Study, a population-based prospective cohort in individuals aged 45 years and over [median 64.1 years (95%-range 49.0–82.8)]. Dietary intake was assessed at baseline with a food-frequency questionnaire. For all participants, we examined adherence (yes/no) to fourteen items of the guidelines: vegetables (≥200 g/day), fruit (≥200 g/day), whole-grains (≥90 g/day), legumes (≥135 g/week), nuts (≥15 g/day), dairy (≥350 g/day), fish (≥100 g/week), tea (≥450 mL/day), ratio whole-grains:total grains (≥50%), ratio unsaturated fats and oils:total fats (≥50%), red and processed meat (<300 g/week), sugar-containing beverages (≤150 mL/day), alcohol (≤10 g/day) and salt (≤6 g/day). Total adherence was calculated as sum-score of the adherence to the individual items (0–14). Information on disease incidence and all-cause mortality during a median follow-up period of 13.5 years (range 0–27.0) was obtained from data collected at our research center and from medical records. Using Cox proportional-hazards models adjusted for confounders, we observed every additional component adhered to was associated with a 3% lower mortality risk (HR 0.97, 95% CI 0.95; 0.98), lower risk of stroke (HR 0.95, 95% CI 0.92; 0.99), chronic obstructive pulmonary disease (HR 0.94, 95% CI 0.91; 0.98), colorectal cancer (HR 0.90, 95% CI 0.84; 0.96), and depression (HR 0.97, 95% CI 0.95; 0.999), but not with incidence of coronary heart disease, type 2 diabetes, heart failure, lung cancer, breast cancer, or dementia. These associations were not driven by any of the individual dietary components. To conclude, adherence to the Dutch dietary guidelines was associated with a lower mortality risk and a lower risk of developing some but not all of the chronic diseases on which the guidelines were based.

**Electronic supplementary material:**

The online version of this article (doi:10.1007/s10654-017-0295-2) contains supplementary material, which is available to authorized users.

## Introduction

In order to prevent chronic diseases in the general population, the Health council of the Netherlands recently presented new national food-based dietary guidelines [1]. These guidelines were developed on the basis of evidence from 29 systematic reviews of prospective cohort and interventional studies on effects of nutrients, foods and dietary patterns on risk of major chronic diseases [2]. For their review, ten major diet-related diseases were taken into account—on the basis of mortality, life-years lost, and burden of disease: coronary heart disease (CHD), stroke, heart failure (HF), type 2 diabetes mellitus (T2DM), chronic obstructive pulmonary disease (COPD), breast cancer, colorectal cancer, lung cancer, cognitive decline, and depression. Following the existing evidence as reported in systematic reviews, a general dietary advice was formulated to consume less animal-based foods and follow a more plant-based dietary pattern [2]. In addition, 15 more specific guidelines were presented on the consumption of fruits and vegetables, fish, legumes, nuts, dairy, whole-grain products, fats and oils, tea, coffee, refined cereals, red meat, sugar-containing beverages, alcoholic beverages, and salt; and a separate guideline for dietary supplements [2].

Although the guidelines are based on extensive previous research on nutrients, foods and dietary patterns in relation to specific diseases, the association of adherence to these overall dietary guidelines with chronic diseases has not yet been evaluated. As effects of overall diet may be different from the sum of the individual foods and nutrients it constitutes [3], the overall diet resulting from following these guidelines also requires evaluation. Therefore, we aimed to examine the criterion validity of the 2015 Dutch Dietary Guidelines by examining the association of adherence to the guideline with all-cause mortality and with the incidence of the ten chronic diseases on which the guidelines were based, in a large population of Dutch middle-aged and older people.

## Methods

### Study design and population

This study was embedded in three sub-cohorts of the Rotterdam Study (RS), a population-based prospective cohort in Rotterdam, the Netherlands. Details of the study design and participants are described elsewhere [4]. Briefly, the first sub-cohort (RS-I) was established in 1990, and all residents aged 55 years and over living the Ommoord district of Rotterdam were invited to participate. Of the 10,215 eligible individuals, 7983 participated (78.1%). In the year 2000, the study was extended with a second sub-cohort (RS-II) of 3011 participants (out of 4472 invited, 67.3%) who had moved into the area or who had become 55 years of age since the start of the study. A third sub-cohort (RS-III) was established in 2006, in which 3932 participants aged 45 years and over were included (out of 6057 invitees, 64.9%). All participants visited the research center at baseline and subsequently every 3 to 5 years for detailed follow-up measurements.

The Rotterdam Study has been approved by the medical ethics committee according to the Wet Bevolkingsonderzoek ERGO (Population Study Act Rotterdam Study), executed by the Ministry of Health, Welfare and Sport of the Netherlands, and written informed consent was obtained from all study participants.

**Fig. 1:**
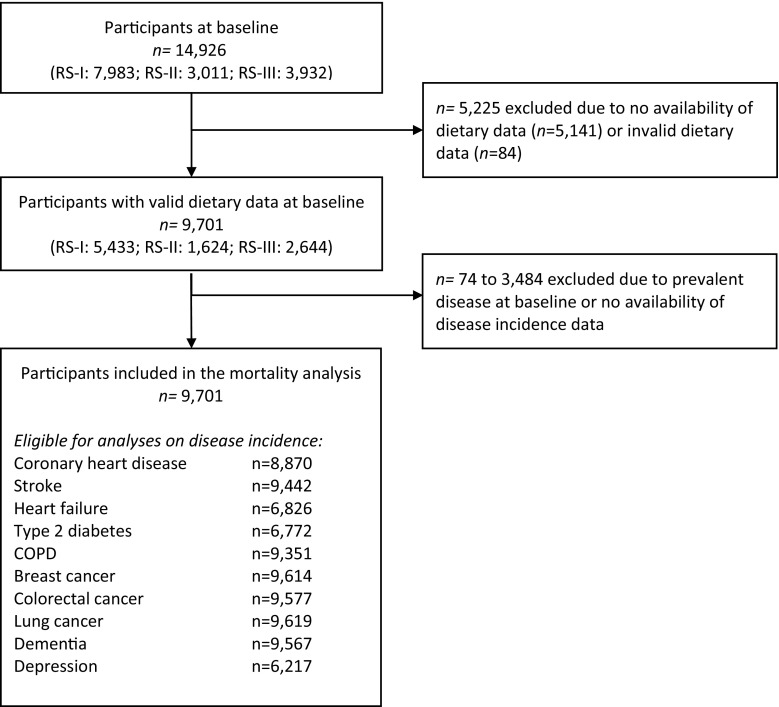
Flow-chart

For the current analysis, we included all participants with reliable dietary data at baseline n = 9701; 5433 from RS-I, 1624 from RS-II, and 2644 from RS-III; Fig. 1). Per analysis, for each of the ten diseases, prevalent cases of that particular disease and participants with incomplete follow-up data on incidence on the disease were excluded, resulting in a population for analysis ranging from 6217 (for depression) to 9627 (for cancer) participants for the disease incidence analyses.

### Dietary assessment

Dietary intake was assessed at baseline using validated food frequency questionnaires (FFQ). A slightly different approach was applied to the first two cohorts of the Rotterdam Study (RS-I and RS-II) than to the third cohort (RS-III). For the first two cohorts, an FFQ was applied in a two-stage approach. In the first stage, participants indicated which foods they consumed at least twice a month in the preceding year using a self-administered checklist of 170 food items. In a second stage, a trained dietician used this list to identify how often and in which amounts the foods were consumed. This FFQ was validated against fifteen 24 h food records and four 24 h urinary urea excretion samples in a subsample of the Rotterdam Study (n = 80), which demonstrated that it was able to adequately rank participants according to their intake: Pearson’s correlation for nutrient intakes with the food records ranged between 0.44 and 0.85 and Spearman’s correlation for protein intake against urinary urea was 0.67 [4]. For the third cohort, a self-administered semi quantitative FFQ was used to assess dietary intake. This FFQ was based on 389 items and was previously validated in two other Dutch populations using a 9-day dietary record [5] and a 4 week dietary history [6], which showed Pearson’s correlations for intakes of different nutrients varying from 0.40 to 0.86. For each food item, the frequency of consumption (in times per month or per week), the number of servings per day (expressed in standardized household measures) as well as the preparation methods were included. Information on portion size, type of food item, and preparation method were collected. Nutrient data were calculated from the Dutch Food Composition Table, using 1993’s version for RS-I, the 2001’s version for RS-II, and 2011’s update for RS-III to account for the changes in nutritional composition of foods. We excluded participants who had an unreliable dietary intake according to the trained nutritionist who performed the interview or because their estimated daily energy intake was implausible, for which cut-offs were set at <500 or >5000 kcal/day.

### Adherence to the Dutch dietary guidelines

The Dutch Dietary Guidelines 2015 describe a general advice to follow a dietary pattern with more plant-based and less animal-based food and further specified recommendations for 15 food groups and for the use of dietary supplements ([1] and Supplemental Table 1). Briefly, they recommend to consume sufficient vegetables and fruits (≥200 g/day for both), whole-grain products (≥90 g/day), legumes (at least once weekly), unsalted nuts (≥15 g/day), fish (1 serving/week); and tea (3 cups/day); to replace refined cereal products by whole-grain products; to replace butter, hard margarines and cooking fats by soft margarines, liquid cooking fats and vegetable oil; and to replace unfiltered coffee by filtered coffee; and to limit the use of red meat, particularly processed meat (quantity unspecified); sugar-containing beverages; alcohol (none or ≤1 glass/day); and salt (≤6 g/day). The use of nutrient supplements is not advised except for people who belong to a group to which specific supplementation advice applies. From these items, we were unable to evaluate filtered coffee and dietary supplement use in our study population because we had no complete information on these items. For the remaining 14 items, we further quantified the guidelines, using additional information from the Netherlands Nutrition Center and Dutch food consumption surveys for the items where no specific cut-offs were provided in the original guideline (Supplemental Table 1). Subsequently, we scored every participant as adhering to this item (‘yes’ scored as 1) or not adhering to the item (‘no’ scored as 0; Table 1). As overall measure of adherence to the guidelines, we used the sum of the number of items adhered to, with a theoretical range from 0 (no adherence) to 14 (full adherence).

**Table 1:** Baseline characteristics and adherence to the dietary guidelines (n = 9701*)*

	Median (95% range) or percentage
Age (years)	64.1 (49.0–82.8)
Gender (% female)	58.1
Educational level (%)^a^	
Primary	15.6%
Lower	41.1%
Intermediate	27.9%
Higher	15.5%
Paid employment (%)	27.7%
Smoking status (%)^a^	
Never	32.1%
Ever	44.2%
Current	23.8%
BMI (kg/m^2^)^a^	26.3 (20.3–36.4)
Physical activity (METh/week)^a^	
RS-I and II, Zutphen questionnaire (n = 7057)	76.6 (14.5–186.9)
RS-III, LASA questionnaire (n = 2644)	42.0 (2.6–200.9)
*Dietary characteristics*	
Energy intake (kcal/day)	2089 (1155–3489)
Number of items adhered to (no.)	7 (3–10)
Adherence to individual guidelines components (%)	
Vegetables ≥200 g/day	48.9%
Fruit ≥200 g/day	54.0%
Whole grain products ≥90 g/day	70.0%
Legumes ≥135 g/week^b^	14.3%
Nuts ≥15 g/day	17.0%
Dairy ≥350 g/day	47.1%
Fish ≥100 g/week	32.9%
Tea ≥450 mL/day	30.0%
Whole grains ≥50% of total grains	82.2%
Unsaturated fats and oils ≥50% of total fats	73.7%
Red and processed meat <300 g/week	12.8%
Sugar-containing beverages ≤150 mL/day	82.9%
Alcohol ≤10 g/day	60.8%
Salt ≤6 g/day	60.9%

^a^Values are based on imputed data. Number of missings per variable were 56 for educational level; 123 for BMI; 1819 for physical activity; and 46 for smoking status

^b^Fresh weight

### Mortality and disease incidence

#### Mortality

Information on vital status of participants was obtained on a weekly basis via municipal population registries and through general practitioners’ and hospitals’ databases. Events were coded according to the International Classification of Diseases 10th version (ICD-10) by two independent research physicians. All-cause mortality was defined as participants who died from any cause during the total follow-up period, which was completed until June 2017.

#### Coronary heart disease (CHD)

CHD was defined as fatal or nonfatal myocardial infarction (MI), or definite coronary mortality. Information on the collection of cardiac outcomes in the Rotterdam Study has been described in detail elsewhere [7]. At baseline, history of MI was assessed by interview and verified using medical records. Participants were monitored for incident CHD by continuous linkage to files from general practitioners in the study area, information from medical specialists and discharge reports after hospitalization. This information was independently reviewed by two study physicians, supervised by a medical specialist. A validation study (n = 100) of the clinical follow-up event registration of incident MI cases in the Rotterdam study, showed that the clinical follow-up system had a 98% case finding of hospitalized MIs [7]. CHD follow-up data were completed until January 2012.

#### Stroke

Stroke was defined in accordance with WHO criteria as a syndrome of rapidly developing clinical signs of disturbance of cerebral function, with symptoms lasting ≥24 h or leading to death and no apparent cause other than of vascular origin. At baseline, history of stroke was assessed by interview and verified using medical records. Subsequently, participants were monitored for occurrence of incident stroke, by continuous digital linkage of the general practitioners’ medical records with the study database, information from medical specialists and discharge reports after hospitalization. This information was reviewed by two independently working study physicians, supervised by an experienced neurologist [8]. Stroke follow-up was completed until January 2014.

#### Heart failure (HF)

Heart failure was defined in accordance with the guidelines of the European Society of Cardiology, requiring objective evidence of cardiac dysfunction, together with typical symptoms of heart failure such as breathlessness, ankle swelling, pulmonary crepitation, or use of cardiovascular medication for HF [9]. At enrollment, prevalent HF was assessed by interview and verified with medical records. Information on incident HF was obtained by continuous linkage to files from general practitioners in the study area, information from medical specialists and discharge reports after hospitalization. This information was independently reviewed by two study physicians, supervised by a medical specialist, and only definite and probable HF diagnoses were included in the analyses [7]. HF follow-up data were completed for the first two cohorts only, until March 2010.

#### Type 2 diabetes mellitus (T2DM)

Prevalent T2DM was defined as having serum glucose concentrations >11.1 mmol/L or using glucose-lowering drugs at baseline. Incident T2M was defined in accordance with the guidelines of the WHO as having a fasting blood glucose concentration ≥7.0 mmol/L, having a non-fasting blood glucose concentration of ≥11.1 mmol/L, or using of blood glucose-lowering drugs on which information was obtained from both home interviews and pharmacy dispensing records. Because no fasting blood samples were collected in the first two visits of RS-I, we set the third visit (1997–1999) of RS-I as baseline, leading to a smaller sample size for T2DM incidence analyses (*n* = 6772). All potential T2DM events were reviewed independently by two study physicians, supervised by an endocrinologist as described elsewhere [10]. T2DM follow-up data were completed until January 2012.

#### Chronic obstructive pulmonary disease (COPD)

COPD was diagnosed according to the GOLD guidelines when the ratio of forced expiratory volume in 1 s (FEV1) over the forced vital capacity (FVC) was below 0.70 [11]. In absence of an interpretable spirometry, medical records of all study participants using frequently COPD medication were carefully evaluated and only included to the case set if a clear physician diagnosis of COPD was retained in the medical records. Information on the validation of COPD in the Rotterdam Study has been described in detail elsewhere [12]. Follow-up data were completed until December 2014.

#### Breast cancer

Breast cancer cases were defined on the basis of the International Classification of Diseases (ICD-10 code C50). The diagnoses of breast cancer were obtained through general practitioners and by linkage with a nationwide registry of histopathology and cytopathology in the Netherlands (PALGA). Two research physicians independently assessed the diagnosis and date of diagnosis of breast cancer. Data on breast cancer incidence were completed until January 2013.

#### Colorectal cancer

Colorectal cancer cases were defined on the basis of the International Classification of Diseases (ICD-10 code C18, C19 and C20). The diagnoses of colorectal cancer were obtained through the general practitioners and by linkage with PALGA. Two research physicians independently assessed the first date and diagnosis of colorectal cancer. Data on colorectal cancer incidence were complete until January 2013.

#### Lung cancer

Lung cancer cases were defined on the basis of the International Classification of Diseases (ICD-10 code C34). The diagnoses of lung cancer were obtained through the general practitioners and by linkage with PALGA. Two research physicians independently assessed the first date and diagnosis of breast cancer. Data on lung cancer incidence were completed until January 2013.

#### Dementia

Participants were screened for dementia using the Mini-Mental State Examination (MMSE) and the Geriatric Mental Schedule (GMS) at baseline and follow-up visits to the research center. If screened positive (MMSE <26 and/or GMS >0), participants underwent an informant interview and examination using the Cambridge Examination for Mental Disorders in the Elderly and further subsequent neurological, neuropsychological and neuroimaging examination if required. In addition, all participants, also those who could not be examined in person at the research center, were monitored for occurrence of incident dementia, by continuous digital linkage of the general practitioners’ medical records with the study database, information from medical specialists, and information from the regional institute for outpatient mental health care [13]. A consensus panel, led by a neurologist, decided on the final diagnosis of dementia. Follow-up for dementia was completed until January 2015.

#### Depression

Data on incident depression were obtained from psychiatric examinations, self-reported histories of depression, medical records, and registration of antidepressant use [14]. The self-reported history of depression included standardized questions to evaluate if and when participants had experienced a depressive episode and, if so, whether they had been treated. Medical records were continuously monitored for potential depressions by trained research assistants. For the psychiatric examinations during the research center visits, participants filled in the Center for Epidemiological Studies Depression Scale questionnaire (CES-D). Positively screened participants underwent a semi-structured clinical interview conducted by a clinician to diagnose depressive disorders. Two research physicians independently decided on the final diagnosis based on all gathered data and discussed discordant assessments. Follow-up data were available for the first two cohorts only, until January 2012. For the current analyses, incident depression was defined as the first event that occurred.

### Covariates

Age at baseline was calculated as the number of years between the date of birth and date of baseline visit to the research center. Information on smoking status and duration was collected through self-report and categorized as never, past, or current smoker. Information on paid employment at baseline (yes/no), and educational level was self-reported at baseline. Educational level was categorized into: primary education with or without a partially completed higher education (primary); lower vocational or lower secondary education (lower); intermediate vocational education and or general secondary (intermediate); or higher vocational or university education (higher). Women’s menopausal status at baseline (post-menopausal, yes/no) was self-reported.

Information on daily energy intake was obtained from the previously described FFQ. Two different questionnaires were used to assess physical activity: For RS-I and RS-II, physical activity was assessed using a validated adapted version of the Zutphen Physical Activity Questionnaire [15]; and for RS-III, physical activity was assessed using the LASA Physical Activity Questionnaire (LAPAQ) [9]. Both questionnaires included questions on walking, cycling, gardening, sports, and housekeeping. Data were recalculated into metabolic equivalent of task (MET)-hours per week for each participant [16]. As different methods were used to estimate physical activity in the different cohorts, MET-hours of the participants were divided into cohort-specific quintiles. At baseline, weight (kg) and height (cm) were measured at the research center, and BMI (kg/m^2^) was calculated.

### Statistical analyses

To assess the association of adherence to the dietary guidelines with mortality risk and incidence of diseases, we used multivariable Cox proportional hazard models. We tested for non-linearity by including a quadratic term of the dietary guideline score in the models. Hazard ratios (HRs) for mortality or disease incidence were calculated per item increase in adherence to the guidelines and for quintiles of adherence to the dietary guidelines with the lowest quintile as reference.

The basic model (model 1) was adjusted for cohort, age at dietary assessment, and sex. The confounder model (model 2) was additionally adjusted for educational level, employment status, smoking status, physical activity, and energy intake. Because we considered BMI to be a potential intermediate in the association of diet quality with disease, we additionally included BMI in a separate model (model 3). We evaluated effect modification by age, sex, total energy intake, and BMI by including the interaction term of the dietary guideline score with the covariable in models 2 and 3. In addition, an interaction term between smoking and the dietary guideline score was evaluated for COPD and lung cancer; and interaction of menopausal status and the diet score was evaluated for breast cancer.

To examine the robustness of our findings, we performed several sensitivity analyses. First, we repeated all analyses in each of the three cohorts separately. Second, we repeated analyses after excluding participants who died (for the mortality analysis) or developed the disease of interest (for disease incidence) within the first two years after follow-up. Third, among women, we additionally adjusted our findings for menopausal status. Fourth, we additionally adjusted for duration of smoking for analyses with COPD and lung cancer as outcomes. Fifth, to check if associations were not driven by one specific guideline for a specific food group, we repeated our main analyses by excluding one of the 14 individual guidelines from the total dietary guideline score one at a time.

To assess the proportion of disease attributable to poor adherence to the 2015 Dutch Dietary Guidelines in the Netherlands, we computed Population Attributable Risks (PAR) for diseases that were significantly associated with adherence to dietary guidelines using the following equation: PAR% = 100 × P_e_(RR − 1)/(P_e_(RR − 1) + 1), where P_e_ is the prevalence of the exposure (i.e., adherence to dietary guidelines). We calculated PAR for less than 25%, less than 50%, or less than 75% adherence to the guidelines.

To reduce potential bias associated with missing data, missing values of covariables were multiple imputed (*n* = 10 imputations) [17], according to the Fully Conditional Specification method (predictive mean matching), assuming no monotone missing pattern. As effect estimates were similar before and after imputation, we only report pooled effect estimates after the multiple imputation procedure. Analyses were performed using SPSS version 21.0 (IBM Corp., Armonk, NY, USA).

## Results

### Population characteristics

Median age of the participants at baseline was 64.1 years (95%-range 49.0–82.8) (Table 1 and Supplemental Table 2). Participants had a median energy intake of 2089 kcal/d (95% range 1155–34,891) and had a median dietary guideline adherence score of 7 (95% range 3–10). None of the participants fully adhered to the guidelines. For the individual dietary guideline items, adherence to the recommendation to limit sugar-containing beverages was high (82.9% of the population), whereas adherence to the recommended legume and nut intake was low (14.3 and 17.0% of the study population, respectively). Characteristics of study participants enrolled in the cohort but without information on dietary intake (n = 5225) are presented in Supplemental Table 3. This group was on average slightly older and more often had a lower educational level as compared to the participants with dietary data (n = 9701).

### Adherence to the dietary guidelines and all-cause mortality risk

Median follow-up time for mortality was 13.5 years (range 0–27.0), during which 4592 out of 9701 participants died (Table 2). After adjustment for confounders (model 2), adherence to the dietary guidelines was associated with a lower mortality risk (HR 0.97 per item adherence; 95% CI 0.95, 0.98). This effect estimate did not change after additional adjustment for BMI (model 3, Table 2). Participants in the highest quintile of adherence to the guidelines had on average a 14% lower risk of dying than participants in the lowest quintile (HR 0.86; 95% CI 0.78, 0.95; *p*-trend over the quintiles <0.001; Table 2).

**Table 2:** Adherence to the dietary guidelines and risk of all-cause mortality

	Basic model (model 1) HR (95% CI)^a^	Confounder model (model 2) HR (95% CI)^a^	Confounder model + BMI (model 3) HR (95% CI)^a^
All-cause mortality (n = 4592 cases/9701 at risk, median FU = 13.5 year (0–27.0)			
Per item higher adherence to the dietary guidelines	0.95 (0.93, 0.96)*	0.97 (0.95, 0.98)*	0.97 (0.95, 0.98)*
Quintile 1	Reference	Reference	Reference
Quintile 2	0.88 (0.76–0.97)*	0.95 (0.86–1.04)	0.95 (0.86–1.04)
Quintile 3	0.81 (0.74–0.89)*	0.93 (0.85–1.01)	0.93 (0.85–1.02)
Quintile 4	0.78 (0.71–0.86)*	0.88 (0.80–0.97)*	0.88 (0.80–0.97)*
Quintile 5	0.78 (0.71–0.86)*	0.86 (0.77–0.95)*	0.86 (0.78–0.95)*
*p*-for-trend^a^	<0.001	<0.001	<0.001

Effect estimates represent hazard ratios (HR) with 95% confidence intervals (95% CI) for all-cause mortality risk per one item higher adherence to the dietary guidelines and for different quintiles of adherence to the dietary guidelines with the lowest quintile as reference

Model 1 is adjusted for cohort, age at dietary assessment, and sex

Model 2 is adjusted for all factors in model 1 and additionally adjusted for smoking status, educational level, employment status, total energy intake, and physical activity

Model 3 is adjusted for all factors in model 2 and additionally adjusted for BMI

* *p* < 0.05

^a^ *p*-for-trend is obtained using the number of the quintiles (i.e., 1, 2, 3, 4, 5) as ordinal variable in the regression model

### Adherence to the dietary guidelines and disease incidence

Median follow-up time for incidence of non-communicable diseases ranged from 7.3 years for T2D to 11.8 years for stroke. Further details on follow-up time and number of cases per disease provided in Table 3. After adjustment for confounders (model 2), adherence to the dietary guidelines was significantly associated with a lower risk of stroke (HR 0.95; 95% CI 0.92, 0.99) and COPD (HR 0.94; 95% CI 0.91, 0.97), but not significantly with risk of HF, T2DM, or dementia (Table 3). Furthermore, higher adherence to the guidelines was also associated with a lower risk of developing colorectal cancer (HR 0.90; 95% CI 0.84, 0.96), but not with breast cancer. Inverse associations of dietary guideline adherence with lung cancer and CHD in basic models (model 1) were driven by confounders since associations were no longer significant in model 2. Finally, adherence to the guidelines was associated with a borderline lower risk of depression (HR 0.97; 95% CI 0.95, 0.999). Additional adjustment for BMI (model 3) did not change the effect estimates for any of the diseases as compared to model 2 (Table 3). Quadratic terms of the dietary guideline score were not significant for any of the outcomes. In line with this, analyses with the dietary guidelines adherence score in quintiles supported findings for the continuous score and associations with stroke, colorectal cancer, COPD, and depression appeared to be approximately linear (Supplemental Table 4).

**Table 3:** Adherence to the dietary guidelines and risk for chronic diseases

	Basic model (model 1) HR (95% CI)^a^	Confounder model (model 2) HR (95% CI)^a^	Confounder model + BMI (model 3) HR (95% CI)^a^
Coronary heart disease n = 1033 cases/8870 at risk Median FU = 10.2 year (0–21.7)	0.96 (0.93–0.99)*	0.98 (0.94–1.01)	0.98 (0.95–1.02)
Stroke n = 979 cases/9442 at risk Median FU = 11.8 year (0–23.7)	0.94 (0.91–0.97)*	0.95 (0.92–0.99)*	0.95 (0.92–0.99)*
Heart failure n = 943 cases/6826 at risk Median FU = 11.7 year (0–19.8)	0.99 (0.96–1.02)	1.00 (0.97–1.04)	1.01 (0.97–1.04)
Type 2 diabetes mellitus n = 642 cases/6772 at risk Median FU = 7.3 year (0–14.7)	0.99 (0.95–1.04)	1.01 (0.97–1.06)	1.03 (0.98–1.07)
COPD n = 1082 cases/9351 at risk Median FU = 11.1 year (0–25)	0.90 (0.87–0.93)*	0.94 (0.91–0.98)*	0.94 (0.91–0.97)*
Breast cancer n = 273 cases/9614 at risk Median FU = 10.9 year (0–22.7)	1.05 (0.98–1.11)	1.04 (0.97–1.12)	1.05 (0.98–1.12)
Colorectal cancer n = 324 cases/9577 at risk Median FU = 11.0 year (0–22.7)	0.90 (0.85–0.96)*	0.90 (0.85–0.96)*	0.90 (0.84–0.96)*
Lung cancer n = 204 cases/9619 at risk Median FU = 11.1 year (0–22.7)	0.87 (0.80–0.94)*	0.93 (0.86–1.01)	0.93 (0.86–1.01)
Dementia n = 1118 cases/9567 at risk Median FU = 11.6 year (0–23.7)	1.01 (0.98–1.04)	1.01 (0.98–1.05)	1.01 (0.98–1.05)
Depression n = 1686 cases/6217 at risk Median FU = 10.9 year (0–18.3)	0.96 (0.94–0.99)*	0.97 (0.95–1.00)*	0.97 (0.95–1.00)*

Model 1 is adjusted for cohort, age at dietary assessment, and sex (exception: sex was not included in the models for breast cancer)

Model 2 is adjusted for all factors in model 1 and additionally adjusted for smoking status, educational level, employment status, total energy intake, and physical activity

Model 3 is adjusted for all factors in model 2 and additionally adjusted for BMI

* *p* value <0.05

^a^Effect estimates represent hazard ratios (HR) with 95% confidence intervals (95% CI) for incidence of developing the disease per one item higher adherence to the dietary guidelines

### Additional analyses

We examined effect modification by age, sex, total energy intake, and BMI for all outcomes. There was a significant interaction of the dietary guideline sore with sex on stroke (*p* = 0.008), but not with any of the other diseases (*p* > 0.20). Stratification by sex showed that the association of adhering to the dietary guidelines with stroke was only present among men (HR 0.90; 95% CI 0.85, 0.95) and not among women (HR 0.99; 95% CI 0.95, 1.04). Further stratification of the women by menopausal status at baseline showed no clear differences in associations of diet quality with stroke between post-menopausal women versus peri-menopausal or pre-menopausal women. We also observed a significant positive interaction between age and adhering to the guidelines for all-cause mortality risk (*p* = 0.03), suggesting a less strong association with older age; and a negative interaction for depression (*p* = 0.02), suggesting stronger associations with older age. Stratification by age groups showed that, indeed, associations of adhering to the guidelines with mortality risk were stronger at younger ages [e.g. HR 0.95 (0.92; 0.99) for <65 years and HR 0.98 (0.96, 0.999) for ≥65 years]; whereas associations with depression were only present at older ages [e.g. HR 1.00 (0.96; 1.05) for <65 years and HR 0.96 (0.92; 1.00) for ≥65 years]. Furthermore, there was a positive interaction of energy intake with the guideline score for depression (*p* = 0.02); and a negative interaction of BMI with the dietary guideline score for breast cancer (*p* = 0.02), but not with any of the other outcomes. We observed no interaction of the dietary guideline score with smoking status for COPD (*p* = 0.64) or for lung cancer (*p* = 0.70); or with menopausal status for breast cancer (*p* = 0.56).

Effect estimates did not differ greatly between the three individual cohorts, although the variation was generally larger in the two youngest cohorts with shorter follow-up time and smaller sample sizes (Supplemental Table 5). Effect estimates of analyses from which we excluded participants who died or developed the disease of interest within the first two years of follow-up were similar to those obtained for the complete study population for all diseases, except for depression (Supplemental Table 6). For diet quality and depression, there was no longer an association after excluding incident depression cases in the first two years (n = 784) from our analyses (HR 0.99; 95% CI 0.96, 1.02; Supplemental Table 6). Additional adjustment for menopause status or for duration of smoking did not affect the results (data not shown). Finally, excluding each of the dietary components of the dietary guideline adherence score one by one resulted in similar associations with incident disease and mortality risk as observed for the total dietary guideline score (data not shown).

Based on the prevalence of adherence to the guidelines in our cohort and observed associations with mortality and disease, we estimated PAR proportions based on our study population. We estimated that when less than half of the dietary guidelines was adhered to (i.e., a score <7), this has most impact on colorectal cancer, with a PAR% of 29.8%, followed by COPD (PAR% = 19.8%) and stroke (PAR: 16.4%) (Supplemental Table 7).

## Discussion

In this large prospective cohort of almost 10,000 middle-aged and older adults, we found that adherence to the 2015 Dutch food-based dietary guidelines was associated with a lower mortality risk and a lower risk of stroke, COPD, depression, and colorectal cancer. However, adherence to the guidelines was not associated with risk of heart failure, type 2 diabetes, breast cancer, lung cancer, or dementia. In our population, adherence to the dietary guidelines was suboptimal, with a median score of 8 out of a maximum of 14 and with none of the participants having a maximum score.

Our results are partly in line with previous findings on the 2006 Dietary Guidelines in The Netherlands [18]. Van Lee et al. [19] developed the Dutch Healthy Diet Index on the basis of dietary recommendations of 2006 which included recommendations regarding the intake of vegetable, fruit, dietary fiber, fish, saturated fatty acids, trans fatty acids, the number of consumption occasions with acidic drinks and foods, and sodium and alcohol intake. Adherence to these dietary guidelines has been associated with a lower all-cause mortality risk but weaker or null results were found for cardiovascular disease, stroke and cancer [18, 20]. In contrast to the previous Dutch Healthy Diet Index and corresponding dietary guidelines from 2006, the 2015 dietary guidelines are completely food-based [2]. The advantage of using a food-based approach is that, instead of individual nutrients, foods may reflect complex synergistic or interaction effects of nutrients, food structure or preparation methods on health [21]. The 2015 Dutch dietary guidelines are unique in this regard, since other recent dietary guidelines from other countries still combine recommendations on both foods and individual nutrients for example those of the US, Australia and Norway [22].

The 2015 Dutch dietary guidelines have been developed on the basis of systematic reviews summarizing the best evidence on foods, nutrients and dietary patterns and the risk of the 10 most common chronic diseases in the Netherlands: cardiovascular disease (including stroke and heart failure), diabetes, breast cancer, colorectal cancer, lung cancer, COPD, dementia and depression as well as cardiometabolic risk factors [2]. In previous years, several other proxies for dietary quality have emerged including the Mediterranean Diet Score (MDS), Healthy Eating Index (HEI), Diet Quality Index (DQI) and the Healthy Diet Index (HDI) which have found to be associated with lower risk of mortality, cardiovascular disease, COPD and some cancers [23]. The current diet score and previously designed quality scores have generally in common that they are characterized by a high intake of vegetables, legumes, fruits and fibers and a low intake of red and processed meat and fatty acids. Nevertheless, there are also important differences. For example, in including dairy intake, including foods and/or nutrients, and in including only healthier or both healthy and unhealthy components. Furthermore, the scoring systems of these indices are very different making it difficult to directly compare the scores.

We observed that the dietary guideline score was inversely associated with all-cause mortality. Participants in the quintile with the highest diet score were on average 12% more likely to survive than those in the lowest quintile, independent of socio-economic indicators, physical activity, energy intake and BMI. Our results are in line with existing evidence regarding adherence to other dietary guidelines and all-cause mortality risk. For example in a large study among U.S. subjects aged 65 years or older, a better HEI score was associated with a lower mortality risk, taking into account other risk factors, such as history of diseases, age, BMI, and smoking [24]. Furthermore, a systematic review of prospective studies concluded that higher adherence to the traditional MDS was associated with higher survival rates [25].

We also observed a risk reduction for other health outcomes including stroke, COPD and colorectal cancer. These results are partly in line with results on the Alternate Healthy Eating Index 2010 (AHEI-2010) [26], which has several similarities with our dietary guideline score (i.e. high consumption of fruits, vegetables, nuts, legumes and whole grains and low consumption of red and processed meat and sugar containing beverages). Several studies confirmed the AHEI-2010 being associated with a lower risk of cardiovascular disease (including stroke) [26, 27] and cancer (including as well as colorectal cancer) [26, 27], as well as COPD [28].

Although in the guideline report for the 2015 Dutch dietary guidelines indicated that recommended dietary patterns have been shown to convincingly reduce the risk of cardiometabolic diseases [2, 24, 25], we only observed an inverse association of the dietary guideline score with stroke, but not with CHD or type 2 diabetes. An explanation of the null findings could be changes in dietary patterns and treatment policies for primary prevention (e.g., with statins) among those at increased risk for cardiovascular disease [29].

We observed that adherence to the guidelines was associated with lower incidence of colorectal cancer but not with breast cancer or lung cancer. This is line with several studies on adherence to the cancer prevention guidelines from the World Cancer Research Fund (WCRF/AICR), based on energy-dense foods and sugary drinks, plant foods, red and processed meat and alcoholic drinks [30]. These WCRF/AICR recommendations have found to be mostly associated with a lower risk of colorectal cancer [30, 31] whereas inconsistent results have been found for breast and lung cancer [30–32], which may be explained by different subtypes of cancer. For example, studies on diet and breast cancer suggest that the strength of the associations may depend on the hormone receptor subtype of breast cancer [33].

We found a significant protective association of a healthy diet on depression. However, after excluding participants who developed depression in the first two years after dietary assessment, this association was no longer present, suggesting that reverse causality may play a role in which dietary intake is affected by symptoms of depression. Another explanation may be that the relation between diet and depression is bidirectional since in a previous study it was found that prior depression was associated with better diet quality at a later time point, while current depression was associated with poorer dietary habits [34]. Further longitudinal measurements of diet are needed to clarify this hypothesis.

We did not find any association between adherence to the dietary guidelines and dementia. So far, studies on dietary patterns and dementia and cognition have shown inconsistent results. Several individual foods such as alcohol, coffee and specific sources of polyunsaturated fatty acids have been associated with dementia [35] but results on overall indices of diet have been inconsistent [23]. It may be speculated that certain individual foods or nutrients (e.g., specific B-vitamins, flavonoids, or fatty acids) have a more important role in the etiology of dementia than overall dietary quality, but also, dementia is an endpoint that is particularly difficult to follow up and further research on diet quality and objectively measured pre-clinical stages of dementia such as brain pathology would be interesting.

### Methodological considerations

We used a large population-based cohort with long term follow-up, information on several important potential confounders and a broad range of accurately measured incidence of diseases to evaluate the most recent dietary guidelines. Incident diseases were identified based on combined information from questionnaires, detailed measurements at our research center, and continuous monitoring of medical records. However, to interpret the findings some limitations need to be considered. First, we used two different FFQs, composed of different numbers of items. For example, the FFQ used in RS-I and RS-II had less detailed items on types of fish and legumes than the FFQ used in RS-III. Although this may have implications when studying specific nutrients, we expect that the FFQs are equally capable of estimating overall food-based dietary quality, since sensitivity analyses showed no major differences in dietary quality between cohorts. Unfortunately, the items in the FFQs did not distinguish between filtered versus unfiltered coffee or salted versus unsalted nuts, which are important distinctions in the new dietary guidelines. This may have led to an underestimation of the magnitude of the associations for the outcomes that have been particularly associated with coffee and salt consumption in the previous literature such as cardiovascular disease, type 2 diabetes and dementia [36–38]. For some food groups, no quantitative cut-offs were provided in the dietary guidelines, e.g. for legumes the guideline is to eat them weekly, and for SCBs the recommendation is to minimize intake. For these components, the authors based their used cut-offs on additional information from the Netherlands Nutrition Center and Dutch food consumption surveys. Different interpretations may have resulted in slightly different cut-off values for these food groups [39].

Although a strength of our study is the use of multiple endpoints to provide a full overview of the construct validity and potential impact of the Dutch dietary guidelines, this consequently required multiple statistical tests, which may have increased the risk of chance findings. Furthermore, although we adjusted for many confounding variables and conducted several sensitivity analyses, conclusions regarding the causality of the observed associations cannot be made. Replication of these analyses in other populations and studies on the associations of diet quality with preclinical disease risk factors, such as hypertension, dyslipidemia, or brain pathologies, are required to provide stronger evidence on potential causality and to better understand underlying pathways.

### Implications

The Dutch food-based dietary guidelines were developed on the basis of evidence for associations of nutrients, foods, and dietary patterns with incidence of major chronic diseases in cohorts and/or randomized controlled trials. We now also show that adherence to these combined dietary guidelines is associated with a lower risk of some, but not all, of these diseases. Implying that the 2015 Dutch dietary guidelines have moderate criterion validity for preventing incidence or major chronic diseases. However, every item higher adherence to the dietary guidelines was associated with a 3% lower all-cause mortality risk and a reduction in risk of stroke, depression, and colorectal cancer, again emphasizing the importance of a healthy diet in lowering risk of several chronic diseases. In our population, we observed that adherence to the dietary guidelines was suboptimal, with a median score of 8 out of a maximum of 14. None of the participants had the maximum score. In line with a recent analyses of dietary intake of 885 Dutch adults, we observed that dietary guideline adherence was particularly low for intake of legumes, nuts, and fish [39]. Although this may partly be explained because e.g. legume intake is a relatively new component in the guidelines, also current legume, nut and fish intake is low among the Dutch population, suggesting there is plenty of opportunity to improve dietary quality in the Netherlands [2]. Poor adherence to the dietary guidelines attributed most to colorectal cancer, COPD and stroke with PARs varying from 16 to 30% for following less than 50% of the dietary guidelines. This suggests that many cases of these diseases can be attributed to poor adherence to dietary guideline, and that policies to improve adherence to these new dietary guidelines can have vast implications for public health.

## Conclusions

We found moderate criterion validity for the 2015 Dutch food-based dietary guidelines, as adherence to these guidelines was associated with a lower mortality risk and a lower risk of developing some, but not all, of the chronic diseases on which the guidelines were based. In general, adherence to the guidelines was poor and leaves plenty of opportunities for improvement and interventions.

## Electronic supplementary material

Below is the link to the electronic supplementary material.

Supplementary material 1 (DOCX 34 kb)

## Abbreviations

CHD: Coronary heart disease
CI: Confidence interval
COPD: Chronic obstructive pulmonary disease
HF: Heart failure
HR: Hazard ratio
T2DM: Type 2 diabetes mellitus
