# Supplementary material for: Adherence to the 2015 Dutch dietary guidelines and risk of non-communicable diseases and mortality in the Rotterdam Study
Source: Eur J Epidemiol. 2017 Aug 19;32(11):993–1005. doi: 10.1007/s10654-017-0295-2 (PMC5684301; doi:10.1007/s10654-017-0295-2)
Supplement: Supplementary file 1 — Supplementary material 1 (DOCX 34 kb) [file 10654_2017_295_MOESM1_ESM.docx]

Supplementary Material

Supplemental Table 1. Items of the 2015 Dutch Dietary guidelines and operationalization for evaluation in the Rotterdam Study

| **Recommendation in the 2015 Dutch Dietary guidelines** | **Operationalization for evaluation in the Rotterdam Study** |
| --- | --- |
| Eat at least 200 g of vegetables and at least 200 g of fruit daily | Vegetables ≥200 g/d |
| Eat at least 200 g of vegetables and at least 200 g of fruit daily | Fruit ≥200 g/d |
| Eat at least 90 g brown bread, whole-meal bread or other wholegrain products daily | Whole grain products ≥90 g/d |
| Eat legumes weekly | Legumes ≥135 g/wk ^a^ |
| Eat at least 15 g of unsalted nuts daily | Nuts ≥15 g/d ^b^ |
| Eat one serving of fish, preferably oily fish, weekly | Fish ≥100 g/wk ^a^ |
| Drink three cups of tea daily | Tea ≥ 450 mL/d ^a^ |
| Take a few portions of dairy produce daily | Dairy ≥350 g/d ^a,c^ |
| Replace refined cereal products by whole-grain products | Whole grains ≥50% of total grains |
| Replace butter, hard margarines and cooking fats by soft margarines, liquid cooking fats and vegetable oils. | Unsaturated fats and oils ≥50% of total fats |
| Replace unfiltered coffee by filtered coffee | Not used |
| Limit the consumption of red meat, particularly processed meat | Red and processed meat <300 g/wk ^a,d^ |
| Minimize the consumption of sugar-containing beverages | Sugar-containing beverages <150 mL/d ^a,d^ |
| Do not drink alcohol or no more than one glass daily | Alcohol <10 g/d ^a^ |
| Limit salt intake to 6 g daily. | Salt ≤6 g/d |
| Nutrient supplements are not needed, except for people who belong to a group for which supplementation applies | Not used |

*^a^ Based on standard portion sizes and additional information from the Netherlands Nutrition Center*

*^b^ Including salted nuts*

*^c^ Defined as three portions, on the basis of mean intakes in the Dutch population and the recommendation to maintain current consumption [*[*2*](#_ENREF_2)*]*

*^d^ Defined as less than one serving*

Supplemental Table 2. Baseline characteristics and adherence to the dietary guidelines per cohort

|  | **Median (95% range) or percentage** | | |
| --- | --- | --- | --- |
|  | **RS-I**  **1990-1993**  **(n=5433)** | **RS-II**  **2000-2001**  **(n=1624)** | **RS-III**  **2006-2008**  **(n=2644)** |
| Age (y) | 66.8 (55.8-83.9) | 61.5 (55.9-82.9) | 57.1 (47.4-75.6) |
| Gender (% female) | 59.1 | 54.1 | 58.3 |
| Educational level (%)^a^ |  |  |  |
| - Primary | 20.7% | 7.7% | 9.8% |
| - Lower | 42.7% | 45.9% | 34.9% |
| - Intermediate | 28.0% | 28.6% | 27.2% |
| - Higher | 8.6% | 17.8% | 28.1% |
| Paid employment (%) | 12.1% | 24.5% | 61.3% |
| Smoking status (%) ^a^ |  |  |  |
| - Never | 33.5% | 28.9% | 31.1% |
| - Ever | 43.0% | 47.6% | 44.4% |
| - Current | 23.5% | 24.5% | 24.5% |
| BMI (kg/m^2^) ^a^ | 26.0 (20.1-34.6) | 26.9 (20.3-36.4) | 26.9 (20.5-38.8) |
| Physical activity (METh/wk) | 76.8 (14.1-192.0)  (Zutphen) | 76.1 (15.6-169.3)  (Zutphen) | 42.0 (2.6-200.9)  (LASA) |
| **Dietary characteristics** |  |  |  |
| Energy intake (kcal/d) | 1922  (1167-3085) | 2069  (1234-3543) | 2205  (1075-4005) |
| Number of items adhered to (no.) | 7 (3-10) | 6 (3-10) | 7 (3-11) |
| Adherence to individual guidelines components (%) |  |  |  |
| - *Vegetables ≥200 g/d* | 49.9% | 40.6% | 52.0% |
| - *Fruit ≥200 g/d* | 56.5% | 38.5% | 58.4% |
| - *Whole grain products ≥90 g/d* | 69.1% | 76.2% | 68.2% |
| - *Legumes ≥135 g/wk* | 5.0% | 18.1% | 31.0% |
| - *Nuts ≥15 g/d* | 13.1% | 14.9% | 26.1% |
| - *Dairy ≥350 g/d* | 50.7% | 47.7% | 39.4% |
| - *Fish ≥100 g/wk* | 21.5% | 35.0% | 55.0% |
| - *Tea ≥ 450 mL/d* | 38.0% | 37.5% | 8.9% |
| - *Whole grains ≥50% of total grains* | 82.5% | 87.8% | 78.3% |
| - *Unsaturated fats &oils ≥50% of total fats* | 57.8% | 28.6% | 71.8% |
| - *Red and processed meat <300 g/wk* | 9.7% | 10.1% | 20.7% |
| - *Sugar-containing beverages ≤150 mL/d* | 91.0% | 64.5% | 77.6% |
| - *Alcohol ≤10 g/d* | 65.3% | 54.4% | 55.4% |
| - *Salt ≤6 g/d* | 66.9% | 50.2% | 55.2% |

^a^ Values are based on imputed data. Number of missings per variable was 56 for educational level; 123 for BMI; 1,819 for physical activity; and 46 for smoking status.

Supplemental Table 3. Baseline characteristics of participants with versus those without dietary data

|  | **Included in current analysis**  **(n=9,701)** | **Not included due to missing dietary data**  **(n=5,225)** |
| --- | --- | --- |
| Age (y) | 64.1 (49.0-82.8) | 67.7 (48.3-92.1) |
| Gender (% female) | 58.1 | 61.1 |
| Educational level (%)^a^ |  |  |
| - Primary | 15.6% | 25.0 % |
| - Lower | 41.1% | 37.2 % |
| - Intermediate | 27.9% | 24.4 % |
| - Higher | 15.5% | 13.3 % |
| Paid employment (%) | 27.7% | 24.4% |
| Smoking status (%) ^a^ |  |  |
| - Never | 32.1% | 35.2 % |
| - Ever | 44.2% | 39.3 % |
| - Current | 23.8% | 25.6 % |
| BMI (kg/m^2^) ^a^ | 26.3 (20.3-36.4) | 26.6 (19.5-37.4) |
| Physical activity (METh/wk)   - RS-I and II, Zutphen questionnaire - RS-III, LASA questionnaire | 76.6 (14.5-186.9)  42.0 (2.6-200.9) | 67.6 (6.7-167.1)  43.5 (2.2-259-2) |

*Values are median (95%-range) or valid percentages.*

Supplemental Table 4. Adherence to the dietary guidelines in quintiles and risk for chronic diseases

|  | **Confounder model + BMI**  **(model 3)**  HR (95%CI) ^a^ |
| --- | --- |
| **Coronary heart diseases** |  |
| Per item higher adherence to the dietary guidelines | 0.98 (0.95-1.02) |
| - Quintile 1 | *reference* |
| - Quintile 2 | 0.93 (0.78, 1.12) |
| - Quintile 3 | 0.88 (0.73, 1.06) |
| - Quintile 4 | 0.97 (0.80, 1.17) |
| - Quintile 5 | 0.92 (0.75, 1.13) |
| P-for-trend ^b^ | 0.52 |
| **Stroke** |  |
| Per item higher adherence to the dietary guidelines | 0.95 (0.92-0.99)* |
| - Quintile 1 | *reference* |
| - Quintile 2 | 0.87 (0.72, 1.05) |
| - Quintile 3 | 0.88 (0.73, 1.06) |
| - Quintile 4 | 0.92 (0.76, 1.11) |
| - Quintile 5 | 0.74 (0.60, 0.91)* |
| P-for-trend ^b^ | 0.02 |
| **Heart failure** |  |
| Per item higher adherence to the dietary guidelines | 1.01 (0.97-1.04) |
| - Quintile 1 | *reference* |
| - Quintile 2 | 1.06 (0.87, 1.28) |
| - Quintile 3 | 0.94 (0.77, 1.15) |
| - Quintile 4 | 1.07 (0.87, 1.31) |
| - Quintile 5 | 1.06 (0.85, 1.31) |
| P-for-trend ^b^ | 0.65 |
| **Type 2 diabetes mellitus** |  |
| Per item higher adherence to the dietary guidelines | 1.03 (0.98-1.07) |
| - Quintile 1 | *reference* |
| - Quintile 2 | 1.01 (0.79, 1.29) |
| - Quintile 3 | 1.18 (0.93, 1.49) |
| - Quintile 4 | 0.98 (0.76, 1.26) |
| - Quintile 5 | 1.20 (0.94, 1.55) |
| P-for-trend ^b^ | 0.24 |
| **Chronic obstructive pulmonary diseases** |  |
| Per item higher adherence to the dietary guidelines | 0.94 (0.91-0.97)* |
| - Quintile 1 | *reference* |
| - Quintile 2 | 0.89 (0.75, 1.06) |
| - Quintile 3 | 0.86 (0.73, 1.03) |
| - Quintile 4 | 0.89 (0.74, 1.07) |
| - Quintile 5 | 0.72 (0.57, 0.87)* |
| P-for-trend ^b^ | 0.004 |
| **Breast cancer** |  |
| Per item higher adherence to the dietary guidelines | 1.05 (0.98 – 1.12) |
| - Quintile 1 | *reference* |
| - Quintile 2 | 0.81 (0.54, 1.12) |
| - Quintile 3 | 0.97 (0.67, 1.40) |
| - Quintile 4 | 0.96 (0.65, 1.40) |
| - Quintile 5 | 0.82 (0.56, 1.19) |
| P-for-trend ^b^ | 0.55 |
| **Colorectal cancer** |  |
| Per item higher adherence to the dietary guidelines | 0.90 (0.84 – 0.96)* |
| - Quintile 1 | *reference* |
| - Quintile 2 | 0.74 (0.54, 1.01) |
| - Quintile 3 | 0.62 (0.44, 0.85)* |
| - Quintile 4 | 0.48 (0.33, 0.69)* |
| - Quintile 5 | 0.71 (0.51,0.99)* |
| P-for-trend ^b^ | 0.002 |
| **Lung cancer** |  |
| Per item higher adherence to the dietary guidelines | 0.93 (0.86 – 1.01) |
| - Quintile 1 | *reference* |
| - Quintile 2 | 1.22 (0.85, 1.76) |
| - Quintile 3 | 1.02 (0.68, 1.51) |
| - Quintile 4 | 0.68 (0.45, 1.06) |
| - Quintile 5 | 0.72 (0.43, 1.21) |
| P-for-trend ^b^ | 0.13 |
| **Dementia** |  |
| Per item higher adherence to the dietary guidelines | 1.01 (0.98-1.05) |
| - Quintile 1 | *reference* |
| - Quintile 2 | 1.07 (0.89, 1.30) |
| - Quintile 3 | 0.99 (0.83, 1.20) |
| - Quintile 4 | 1.04 (0.86, 1.25) |
| - Quintile 5 | 1.10 (0.91, 1.33) |
| P-for-trend ^b^ | 0.49 |
| **Depression** |  |
| Per item higher adherence to the dietary guidelines | 0.97 (0.95-0.999)* |
| - Quintile 1 | *reference* |
| - Quintile 2 | 1.13 (0.98, 1.31) |
| - Quintile 3 | 0.93 (0.80, 1.09) |
| - Quintile 4 | 0.97 (0.83, 1.13) |
| - Quintile 5 | 0.89 (0.76, 1.04) |
| P-for-trend ^b^ | 0.03 |

^a^ *Effect estimates represent hazard ratios (HR) with 95% confidence intervals (95%CI) for incidence of developing the disease per one item higher adherence to the dietary guidelines or for different quintile of adherence to the guidelines as compared to the lowest quintile.*

Models are adjusted for age, sex, smoking status, educational level, employment status, total energy intake, physical activity, and BMI (model 3)

^b^ p-for-trend was calculated by including the numbers of the quintiles (i.e., 1, 2, 3, 4, 5) as ordinal variable in the model.

*p-value<0.05

Supplemental Table 5. Adherence to the dietary guidelines and risk for chronic diseases and mortality in the three cohorts separately

|  | **RS-I**  **(n=5433) ^a^**  HR (95%CI) ^b^ | **RS-II**  **(n=1624) ^a^**  HR (95%CI) ^b^ | **RS-III**  **(n=2644) ^a^**  HR (95%CI) ^b^ |
| --- | --- | --- | --- |
| All-cause mortality | 0.97 (0.95-0.99) | 0.98 (0.93-1.03) | 0.90 (0.82-0.98) |
| Coronary heart diseases | 0.98 (0.94-1.02) | 1.01 (0.91-1.11) | 0.98 (0.83-1.16) |
| Stroke | 0.95 (0.91-0.99) | 0.96 (0.86-1.07) | 1.08 (0.89-1.31) |
| Heart failure | 1.00 (0.96-1.04) | 1.09 (0.94-1.27) | NA |
| Type 2 diabetes mellitus | 1.05 (0.98-1.11) | 0.99 (0.90-1.09) | 1.02 (0.91-1.15) |
| Chronic obstructive pulmonary diseases | 0.91 (0.87-0.95) | 0.96 (0.88-1.05) | 0.97 (0.89-1.06) |
| Breast cancer | 1.03 (0.96-1.12) | 1.11 (0.93-1.35) | 1.04 (0.82-1.32) |
| Colorectal cancer | 0.95 (0.88-1.01) | 0.71 (0.59-0.84) | 0.74 (0.55-0.98) |
| Lung cancer | 0.95 (0.87-1.03) | 0.85 (0.67-1.07) | NA |
| Dementia | 1.01 (0.97-1.05) | 1.06 (0.95-1.19) | NA |
| Depression | 0.96 (0.93-0.99) | 1.03 (0.97-1.09) | NA |

**^a^** *overall n, number for mortality analyses, number of participants for disease analyses differs per outcome*

^b^ *Effect estimates represent hazard ratios (HR) with 95% confidence intervals (95%CI) for incidence of developing the disease per one item higher adherence to the dietary guidelines.*

Models are adjusted for age, sex, smoking status, educational level, employment status, total energy intake, physical activity, and BMI (model 3)

*NA: no follow-up data available or number of cases <10*

Supplemental Table 6. Adherence to the dietary guidelines and risk for chronic diseases and mortality, excluding incident cases in the first two years

|  | **Confounder model + BMI**  **(model 3)**  HR (95%CI) ^a^ |
| --- | --- |
| Mortality  *n=4245/9504*  *Median FU=13.3 y (2-26)* | 0.97 (0.95, 0.98)* |
| Coronary heart diseases  *n=904/8536*  *Median FU=10.0 y (2-21.7)* | 0.98 (0.94-1.02) |
| Stroke  *n=892/9185*  *Median FU=12.2 y (0-23.7)* | 0.95 (0.91-0.98)* |
| Heart failure  *n=841/6588*  *Median FU=12.3 y (2-19.8)* | 1.01 (0.97-1.05) |
| Type 2 diabetes mellitus  *n=545/6511*  *Median FU=8.2 y (2-14.7)* | 1.03 (0.98-1.07) |
| Chronic obstructive pulmonary disease  *n=877/5925*  *Median FU=14.1 (2-22.7)* | 0.94 (0.90-0.97)* |
| Breast cancer  *n=195/9481*  *Median FU= 11.0 y (2-25)* | 1.03 (0.95 – 1.10) |
| Colorectal cancer  *n=208/9496*  *Median FU=11.0 y (2-25)* | 0.88 (0.82 – 0.94)* |
| Lung cancer  *n=221/9627*  *Median FU=11.0 y (2-25)* | 0.93 (0.86 – 1.01) |
| Dementia  *n=1118/9567*  *Median FU=12.1 y (2-23.7)* | 1.01 (0.98-1.05) |
| Depression  *n=1058/5433*  *Median FU=11.4 (2-18.3)* | 0.99 (0.96-1.02) |

*^a^ Effect estimates represent hazard ratios (HR) with 95% confidence intervals (95%CI) for incidence of developing the disease per one item higher adherence to the dietary guidelines.*

Models are adjusted for age, sex, smoking status, educational level, employment status, total energy intake, physical activity, and BMI (model 3).

Supplemental Table 7: Population attributable risk proportions (PAR%) for different scenarios of adherence to the Dutch Dietary Guidelines

|  | Less than 25% adherence | Less than  50% adherence | Less than  75% adherence |
| --- | --- | --- | --- |
|  | Adherence to <4 of the 14 components | Adherence to <7 of the 14 components | Adherence to <11 of the 14 components |
| **Prevalence (P_e_) in the current study population** | 4.2% | 45.5% | 98.3% |
| **PAR%** |  |  |  |
| All-cause mortality | 0.5% | 9.8% | 28.1% |
| Stroke | 1.9% | 16.4% | 42.7% |
| Colorectal cancer | 1.2% | 29.8% | 64.2% |
| COPD | 0.5% | 19.8% | 48.9% |
| Depression | 0.5% | 9.8% | 28.1% |

PAR proportions were calculated only for diseases that were significantly associated with adherence to dietary guidelines using the following equation: PAR%=100×P_e_(RR−1)/(P_e_(RR–1)+1), where P_e_ is the proportion of the population in each of the exposure groups. PAR%s are calculated based on estimated hazard ratios adjusted for cohort, age at dietary assessment, sex smoking status, educational level, employment status, total energy intake, and physical activity, and BMI; as presented in tables 2 and 3, model 3.
